# Supplementary material for: Communicable Disease Surveillance in South Africa and LMICs: A Systematic Review of Systems, Challenges, and Integration with Environmental Health
Source: Trop Med Infect Dis. 2025 Nov 3;10(11):314. doi: 10.3390/tropicalmed10110314 (PMC12656745; doi:10.3390/tropicalmed10110314)
Supplement: Supplementary file 1 [file tropicalmed-10-00314-s001.zip › tropicalmed-3939376-supplementary.pdf]

Supplementary documents

Figure S1. PRISMA Flow Diagram.

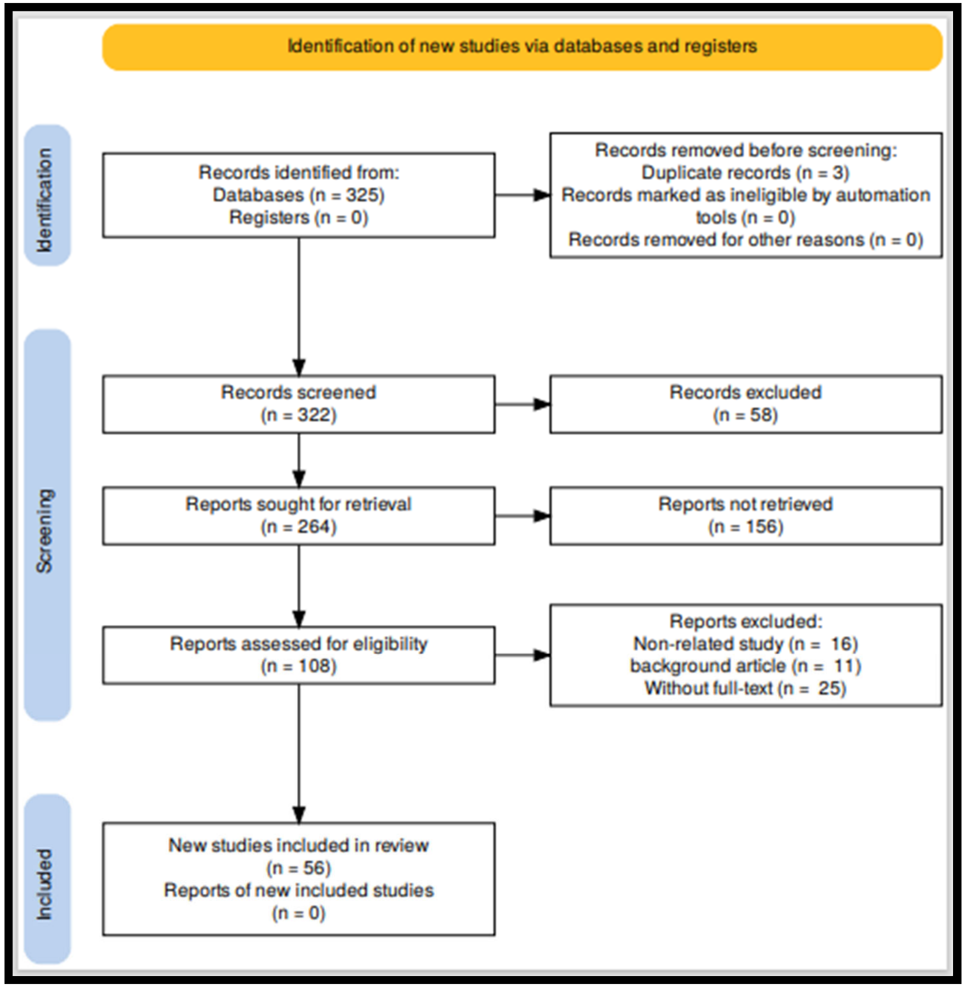

Table S1. AMSTAR 2 Checklist for Quality Assessment.

This checklist was used to critically appraise a systematic review: **Communicable Disease Surveillance in South Africa and LMICs: A Comparative Review of Systems, Challenges, and Integration with Environmental Health.**

Domain No.	Description of Domain	Checklist Item	Response (Yes / No/ Partial)	Comments / Notes
1	Clear review questions (PICO)	Did the research questions and inclusion criteria for the review include the components of PICO?	Yes	Components of PICO were included per PRISMA item 4.
2	Protocol registered before the review	Did the review report contain an explicit statement that the review methods were established before the review, and did the report justify any significant deviations from the protocol?	No	There were no significant deviations from the initially approved protocol.
3	Justification for the study design inclusion	Did the review authors explain their selection of the study designs for inclusion in the review?	Yes	The study considered observational studies relevant to the topic, including previous reviews, cross-sectional studies, exploratory studies, case studies, and comparative studies.
4	Comprehensive literature search strategy	Did the review authors use a comprehensive literature search strategy?	Yes	The study sourced relevant literature using the most reliable databases, including Scopus, Web of Science, PubMed, and Google Scholar.
5	Duplicate study selection	Did the review authors perform study selection in duplicate?	Yes	All duplicates were identified and removed
6	Duplicate data extraction	Did the review authors perform data extraction in duplicate?		The two review authors independently extracted the data using the same standardized data extraction tool.
7	Listing of excluded studies with reasons	Did the review authors provide a list of excluded studies and justify the exclusions?	Yes	The Prisma flow diagram was followed to ensure adherence to the inclusion and exclusion criteria. It therefore indicates the reasons for the excluded studies.
8	Description of included studies	Did the review authors describe the included studies in adequate detail?	Yes	The detailed inclusion criteria were specified to provide greater clarity on which studies to select.
9	Risk of bias (RoB) assessment in individual studies	Did the review authors use a satisfactory technique for assessing the risk of bias (RoB) in individual studies that were included in the review?	Yes	RoB was assessed with reference to items 11 and 18 of the PRISMA 2020 guideline. Therefore, the risk of bias for the included studies was assessed independently by the two review authors using the AMSTAR 2 tool.
10	Reporting the funding sources of included studies	Did the review authors report on the sources of funding for the studies included in the review?	Yes	Although the funding sources are the authors themselves, no additional funding source is acquired.

11	Use of appropriate meta-analytic methods	If meta-analysis was performed, did the review authors use appropriate methods for the statistical combination of results?	Yes	The PRISMA guideline was used.
12	Assessment of the impact of RoB on results	If meta-analysis was performed, did the review authors assess the potential impact of risk of bias in individual studies on the results?	Yes	Two independent reviewers were involved in the analysis process to avoid the risk of bias in individual studies and ensure that subjective bias is reduced.
13	Consideration of RoB when interpreting results	Did the review authors account for the risk of bias in individual studies when interpreting/discussing the results of the review?	Yes	Two independent reviewers were involved in interpreting the results to minimize subjective bias.
14	Explanation of heterogeneity	Did the review authors provide a satisfactory explanation for, and discussion of, any heterogeneity observed in the results of the review?	Yes	The reviewed studies used diverse research designs, from cross-sectional and qualitative assessments to systematic reviews and observational studies.
15	Investigation of publication bias	If they performed quantitative synthesis, did the review authors carry out an adequate investigation of publication bias and discuss its likely impact on the results of the review?	No	Only reliable publications were included in the study.
16	Disclosure of conflicts of interest and funding of review	Did the review authors report any potential sources of conflict of interest, including any funding they received for conducting the review?	Yes	No external funding sources are required for this study.

Table S2. Reviewed Studies for Systematic Review

**Study ID No.:**	**Reference**	**Source / Database**	**Study Title**	**Sample Size**	**Study Design**	**Study Area/ Country**	**Communicable Diseases for surveillance**	**CD surveillance systems and strategies**	**Key findings**	**Recommendations**	**Challenges**
S001	[30]	Google Scholar	Strengthening Disease surveillance capacity at national level across five countries: a	57 participants for in-depth interviews with a range of professionals from governmen	Exploratory Review	5 Countries: Côte d'Ivoire, Ecuador, Madagascar, Namibia, and the Kingdom	COVID-19	WHO's Global genomic surveillance. strategy for pathogens with pandemic and	Identified the need for training, and capacity building as critical for establishing and strengthening NPHI. It also emphasised a clear data sharing norms and standards/ guidelines, clear	Develop national strategies to overcome infrastructural, funding, and workforce challenges. To improve disease surveillance at the national level, national genomic	Inconsistency in disease surveillance capacity. Variations in disease surveillance systems and capacities.

				qualitative study.	t, NPHIs, academic institutions and the private sector		of Saudi Arabia		epidemic potential 2022 – 2032.	surveillance strategies and reporting guidelines. The important role of National Public Health Institute in engaging regional structures that fosters collaborative environments.	surveillance plans and reporting guidelines must be prioritised, along with the creation of standards and norms for data sharing between countries and internationally and the encouragement of collaborative environments.	
S002	[37]	2013	PubMe d	Enhanced surveillance for detection and management of infectious diseases: regional collaboration in the middle East	35 profession als	Review	Middle East countries- MEDICS countries: Jordan, Palestinian , and Israel	Foodbor ne disease surveilla nce (Salmone lla, shigella, and avian influenza prepared ness	IHR Laboratory- Based Enhanced Foodborne Disease Surveillance System	In the future, the consortium plans to extend its laboratory- based surveillance network from Salmonella and Shigella to other enteric pathogens, such as enterotoxigenic, Escherichia coli, Campylobacter jejuni, and selected protozoa and viruses of public health importance in the Middle East.	Construct effective food safety policies aimed at improving food trade an exchange in the region while simultaneously reducing the burden of foodborne disease.	key challenge facing MECIDS is the significant lag time that still exists between the different stages of surveillance data collection and reporting.
S003	[27]		Scopus	Building evidences in Public Health Emergency Preparednes s (“Be PHEP” Project)—a	1415 articles, , and 11 studies were considered	System atic Review	Low- and middle- income countries Worldwide : Uganda, Yemen, Phillipines, Haiti,	Cholera outbreak s, and other infectiou s diseases outbreak s such as malaria,	Different interventions implemente d in low-and middle- income countries to prevent and control	Epidemiological surveillance, case management, WASH service, and oral vaccination was implemented for Cholera outbreak in Haiti, Mozambique, and mathematic	To prevent infectious diseases in LMICs during humanitarian crises, careful planning, adequate resources, and robust collaboration	LMICs lack of resources, adequate infrastructure, standardized method dealing outbreaks,

			systematic review			Thailand, Mozambique, India, Nigeria, South Sudan,	Ebola, Measles,	infectious diseases outbreaks such as vaccination campaigns, epidemiologic surveillance, and integrated health services.	model in Thailand. Phillipines improved response time for climate related disasters through point of care testing and spatial care pathways. Surveillance and rapid response measures. Uganda developed multi-hazard emergency plan. Interventions highlighted the importance and need for multimodal, targeted, collaborative response.	among international organisations, NGOs, and local governments are essential, confirming and supporting efforts already underway.	
S004	[28]	PubMed	The lessons of COVID-19 pandemic for communicable diseases surveillance system in Kurdistan Region of Iraq	14 Key Informants (KIs). Seven in one FGD, and seven in-depth interviews	Descriptive cross-sectional study - qualitative research design	Kurdistan Region of Iraq	COVID-19	CDSS comprises of core activities such as case detection, case registration, case confirmation, reporting, data analysis and interpretation, epidemic preparedness, response and control,	Integration of COVID-19 in the system instead a parallel system was developed, The absence of a standard case definition in health centers and hospitals might jeopardize case detection.	Existing CDSS needs review as it lacked opportunity for epidemic preparedness, timeliness, supervision during pandemic, intersectoral collaboration.	Lack of financial support, staff trainings, cooperation amongst stakeholders, scarce resources, managerial and administrative issues.

									and feedback. Support activities: training, stakeholder collaboration, and supervision.			
S005	[63]	2022	PubMed/NLM database	Assessment of core and support functions of the communicable disease surveillance system in the Kurdistan Region of Iraq	291 health facilities HFs (Primary health care centers and Hospitals) in the Kurdistan region of Iraq	Descriptive cross-sectional study	Kurdistan Region of Iraq	53 communicable diseases included in CDS plan manual	Manual surveillance system included case detection, registration, confirmation, reporting, data management and analysis, outbreak detection, epidemic preparedness, feedback	Core and support functions by health facilities. Case definitions, thresholds, and control measures not included in the system. The manual only available at central and district level while was not available at PHCs and hospitals.	Strengthening the CDSS by reinforcing the surveillance system with constant feedback, supervision, well-trained and motivated staff, technical support, and collaboration between researchers, physicians and all involved in the system.	Lack of surveillance data analysis. Lack of epidemic preparedness 3% and feedback 53%. Standard guidelines and resource allocation 0%. CD control guidelines not reviewed.
S006	[40]		PubMed	Federation of Infectious Diseases Societies of Southern Africa guideline: Recommendations for the detection, management, and prevention	19-member panel for discussion	Guideline	Johannesburg, SA	Candida auris causing candidemia, other candida species.	Laboratory identification and testing. Infection prevention and control measures	18 recommendations provided to cover laboratory identification, testing, surveillance and response, infection prevention and control, clinical management, and antifungal stewardship.	Perform species-level identification for all candida cultured. Nationally coordinated surveillance for *Candida auris* be integrated into broader surveillance for antimicrobial resistance. Regular cross-sectional surveys to monitor	Non-integrated antimicrobial resistant into a broader surveillance system. Environmental screening not appropriately conducted.

			of healthcare-associated Candida auris colonisation and disease in South Africa							epidemiological and environmental trends over time.	
S007	[39]	Google Scholar	The Burden of Infectious Diseases: A Trend Appraisal in Sub-Saharan Africa Regional Trade Blocs.	14 ECOWAS member countries and also 14 member countries of the SADC.	Comparative study	Economic Community of West Africa States (ECOWAS) and the Southern Africa Development Community (SADC) trade blocs	TB and HIV prevalence rates	IDSR system	The magnitude and severity of both diseases were higher in SADC than ECOWAS. Both diseases exceeded the global average in both areas. Less success in reducing the burden of HIV prevalence were recorded in South Africa, Swaziland, Lesotho, and Botswana in 2016. More efforts needed in curbing the infectious diseases in both SADC and ECOWAS. SSA region is a significant contributor to the global burden of infectious diseases.	Intensify the existing efforts at curbing the threat of TB and HIV infections. Awareness campaigns for the prevention and control should be given more attention and programs be in local language. ART be accorded more attention in health policy formulation including adequate budgeting by national governments.	Rapid urbanization, risky culture and sexual behaviours remain the identified challenges that give rise to both infectious diseases in the study areas.

S008	[57]	PubMed	An overview of disease surveillance and notification system in Nigeria and the roles of clinicians in disease outbreak prevention and control	Not specified	Review	Nigeria	40 communicable and non-communicable diseases such as epidemic prone diseases (Cholera, Diarrhoea with blood (Shigella Sd1), Measles, Meningitis, Viral haemorrhagic fevers (Lassa, Ebola Virus Disease), Human influenza caused by a new Subtype, yellow fever, Severe Acute Respiratory	Integrated Diseases Surveillance and Response and disease notification system	Outlined the impact of timely reporting notifiable diseases including the importance and roles of clinicians in disease notification.	The clinicians in Nigeria should be familiar with basic concepts of the IDSR system in order to be prepared to recognize and respond to all disease outbreaks in a timely manner.	Delayed reporting of notifiable diseases by clinicians influenced the emphasise on the roles of clinicians within the system.

							Syndrome (SARS), Smallpox, Dengue fever, Anthrax, Severe Acute Respiratory Illness) and diseases targeted for eradication such as Acute Flaccid Paralysis (AFP)/poliomyelitis, Dracunculiasis, Leprosy, Neonatal tetanus, Lymphatic filariasis, Tuberculosis .				

S009	[31]	PubMed	Notifiable disease reporting among public sector physicians in Nigeria: a cross-sectional survey to evaluate possible barriers and identify best sources of information	245 Physicians	KAP Survey	Six Nigerian cities	Avian Influenza (H5N!) and other infectious diseases	IDSR system	Lack of knowledge amongst 26% of the respondents don't know how and to whom to report infectious diseases	The system should effectively identify AI and other infectious diseases through IDSR, reporting system requirements need to be clearly communicated to the participating physicians and perceived obstacles, such as lack of infrastructure, addressed	Lack of proper infrastructure or logistics and reporting system.
S010	[32]	Google Scholar – grey literature	Communicable disease Surveillance in the City of Ekurhuleni: Environmental Health Practitioners' perceptions.	95 EHPs	Cross-sectional Study	Ekurhuleni Metropolitan Municipality/ South Africa	All communicable diseases	Notifiable medical condition system. Passive, Active and Sentinel surveillance.	Insufficient knowledge by EHPs on communicable disease surveillance systems, hence the inconsistent or non-implementation of disease surveillance function.	Training of EHPs on disease surveillance and development of guidelines to enhance their involvement in disease surveillance. A need for comprehensive approach for CDS by EHPs.	Non-adherence to CD regulations. Lack of capacity and comprehensive approach for CDS by EHPs. Lack of EH standards for CDC in SA.
S011	[2]	Scopus	Integrated Disease Surveillance and Response (IDSR) strategy: status, challenges, and perspectives for the	47 African countries	Comparative study	African Region	All infectious diseases	Community-based surveillance, event-based surveillance. IDSR system	44 African countries implementing IDSR system, 32 had community-based surveillance and achieved timeliness and completeness of report threshold, 35 had event-based surveillance	All countries should enhance IDSR system, and periodically analyse whether any events are missed and the timeliness of the response to all events should be able to incorporate population-based surveillance.	Challenges with event-based surveillance. Enhance IDSR in African countries. Lack of workforce, capacity building. Policy makers not prioritizing IDSR and IHR for funding.

			future in Africa.								
S012	[26]	PubMed	Integrated Disease Surveillance and Response (IDSR) in Malawi: Implementation gaps and challenges for timely alert	648 Data from District health information system from 2014 – 2016. Interviews with 29 frontline health workers, and 7 key informants from IDSR administration at district and national levels.	Mixed-method research study	Malawi	The system assesses all infectious diseases	IDSR system and national disease surveillance system. DHIS2	Major achievement in implementation of indicator-based surveillance and only twelve (12) countries had implemented IDSR. Other guidelines used like HAS and CCM register as tool for surveillance including malaria guidelines, ARI depending on presentation of patient.	Strengthen community-based surveillance to enhance timeliness in response to events and increase case detection. Improvement in information technology infrastructure in Malawi, national standardized EMR system and emerging mHealth technologies can be opportunities for the country to overcome the challenges and improve the surveillance system.	Significant difference IDSR implementation between the technical guideline and the practice. Lack of weekly reports, staff trainings and supervision. Community level case identification was not practised. Lack of adequate infrastructure (lab facilities) and resources as DHIS require data capturing on computer while not all offices had access to such resources. Timeliness is a general problem across African countries resulting in late detection of events

S013	[42]	2024	PubMed	Burden and trends of infectious disease mortality attributed to air pollution, unsafe water, sanitation, and hygiene, and non-optimal temperature globally and in different socio-demographic index regions	21 Regions Retrieved data from global burden of diseases study between 1990 - 2019	Observational study	The Caribbeans, Sub-Saharan Africa, South Asia,	Deaths from Enteric infections, respiratory infections, encephalitis, and meningitis	Global Burden of Disease data system	An increased global infectious disease death from air pollution, unsafe water and sanitation mostly affecting the elderly and children.	Establish targeted interventions and policies that focus on improving environmental quality, bolstering water and sanitation infrastructure, and controlling temperature extremes. Prioritizing the health and well-being of these vulnerable populations is crucial in reducing the burden of infectious diseases and further advancing global public health. Moreover, fortifying international cooperation is key to bridging the disparities across regions, propelling global public health endeavours, and achieving the SDGs	Lack of policies in improving environmental quality. Non-prioritization of health in the vulnerable population. only six individual diseases reported. More interventions required to enhance environmental quality, including extreme temperatures. International cooperation to bridge regional disparities

S014	[1]	PubMed	Infectious disease in an era of global change.	worldwide	Systematic review	Global review: East Asia and Pacific, Europe and Central Asia, Middle East and North Africa, Sub-Sahara Africa, Latin America and Caribbean, North America	TB, HIV/AIDS, NTDs, Malaria, Diarrhoeal infections, Respiratory infections,	Multiple surveillance platforms, Genomic surveillance system, Serological surveys, Artificial intelligent and machine model	Evidence on Climate change significantly increasing the risk of infectious diseases.	Reducing inequities in access to health care and improving surveillance and monitoring for infectious diseases in low- income and middle- income countries, and in underserved populations within countries, should be a priority in tackling pathogen emergence and spread. Increased investment in outbreak response, such as the recent formation of the WHO Hub for Pandemic and Epidemic. collaborative, worldwide framework for infectious disease research and control.	Uneven distribution of available disease data including biases in representative sampling. Inequalities in access to services and surveillance and monitoring of infectious diseases prevalent in LMIC.
S015	[7]	PubMed	Listeriosis outbreak in South Africa: a comparative analysis with previously reported cases worldwide.	6 countries. Considered Data generated during 2017 – 2018 SA outbreak, and major outbreaks in other	Comparative study	Review in SA, US, EU, Australia, Asia, and Nigeria.	Listeriosis, complicate to meningitis, encephalitis, rhombencephalitis, meningo	IDSR system. National notifiable disease system,	SA recorded the highest rate of the outbreak, indicating limitations in food safety policies, and weak CD prevention and control strategies. Austria only had two cases with one fatality, Denmark had four cases with one fatality, while Finland	Strengthen CD prevention and control strategies including food safety policies to improve early detection and response to the outbreaks.	Overall limits on reports, surveillance systems, and food safety legislation.

				parts of the world US, EU, Australia, Asia, and Nigeria.			encephalitis, septicaemia		had 14 cases with only two fatalities. Sweden reported six cases with two fatalities, and UK also had six cases.		
S016	[11]	Google scholar/ PubMed	Environmental surveillance as a tool for identifying high-risk settings for typhoid transmission.	4 regions South Asia, Sub-Saharan Africa, North America and Europe	Studies review	Reviewed relevant Studies in US and European cities	Enteric fever (typhoid fever)	Environmental surveillance. Vaccination program in high-risk area.	surveillance of enteric fever was found to be costly as hybrid surveillance models required for generating population-based incidence estimate. Environmental surveillance	Enhance environmental sampling as a tool for generating actionable data that can inform public health responses to enteric fever. Establish population-based clinical surveillance as a reference standard to enable their interpretation in settings with unknown disease burden.	Lack of typhoid burden data in middle-income countries. Lack of resources in environmental surveillance for disease prevention and control.
S017	[13]	PubMed	An isolated outbreak of diphtheria in South Africa, 2015	15 cases	Case study	KwaZulu Natal, South Africa	Diphtheria disease	District Health Information System.	Majority of cases (75%) occurred in incomplete immunization groups. Other comorbidities were not proved to contribute to the manifestation of cases. Hence the outbreak occurred in the area where HIV infection is highly	Improve continuous immunization coverage of Diphtheria in KZN and ensure available of vaccine stock.	Low coverage of Diphtheria immunization in KZN. Insufficient stock of vaccine

									prevalent but none of cases had HIV infection.		
S018	[35]	PubMed	Outbreak of influenza A in a boarding school in South Africa, 2016	308 students	Retrospective cohort study and cross-sectional study	Eastern Cape, South Africa	Influenza A (H3N2)	NICD data base, medical records.	The cohort study findings highlighted no significant difference in attack rates as only 39/308 (13%) cases occurred. Cross-sectional study findings, 36 cases were identified with attack rate of 20%. Thirty percent of students completed questionnaire were vaccinated 2016 before the outbreak. Concluded that participation in sport squash activities increases the chance of being infected.	Individuals with ILI symptoms should not attend work or school until at least 24 hours after fever subsides. Students, parents, and staff should be educated on the differences between a common cold and influenza to ensure that health care is sought if they experience ILI. Recommended to avoid close contacts; limit visitors; wash hands with soap and water or use an alcohol-based hand rub regularly; and wipe down regularly touched surfaces with a disinfectant. Lastly, it is important to practice good respiratory etiquette by covering the mouth and nose with a tissue when coughing or sneezing. Plan to isolate the symptomatic students	Low influenza vaccine coverage. Missed cases due to restrictions of case definition, as those without cough were excluded. Some cases were clinically diagnosed per medical review record but did not meet the case definition. Non availability of consent forms from parents.

										and staff to avoid the spread.	
S019	[6]	Google Scholar	A Systematic Review of the Integrated Disease Surveillance and Response Implementation Among African Countries Between 2010 and 2024	61 publications	Scoping review	Sub-Saharan Africa	All diseases affecting public health	IDSR	The review indicated significant variations in implementation of IDSR system across Sub-Saharan countries.	National governments should prioritize domestic resource allocation for IDSR activities, ensuring adequate funding for infrastructure, training, and operational costs. National governments should also engage local communities and civil society organizations in IDSR activities to ensure comprehensive coverage and community participation. Decentralized training programs that target district and community health workers can significantly improve disease surveillance at the grassroots level.	Some countries are challenged by inadequate infrastructure, lack of resources, lack of capacity building, poor data management rendering the system ineffective.

S020	[29].	2014	Google Scholar	Challenges of data collection and disease notification in Anambra State, Nigeria	270 HCW	Cross-sectional study	Nigeria	Notifiable diseases	Disease surveillance and notification system	Most HCW 54% not trained on DSN system. Poor funding of the system. All secondary and tertiary health facilities have doctors, while only 22% of primary health facilities had doctors.	Strengthen computerized system to enable effective data collection and disease notification. Ensure training of staff in all facilities to improve prompt notification and response.	The DSN system not computerized only paper-based system affecting timeliness and transportation costs. Uneven distribution of HCW for optimal data collection and notification functions.
S021	[33]		Google Scholar	Communicable diseases surveillance and outbreak investigation in South Africa.		Review Report	South Africa	Tuberculosis, HIV, Syphilis	NICD Epidemiological surveillance-	848 cases from 2008-2012.	Communicable diseases surveillance needs to be strengthened and resourced.	Recognised challenges with human and financial resources.
S022	[34]		Google Scholar	Implementation of a Community-Based Public Model for the Prevention and Control of Communicable Diseases in Migrant Communities in Catalonia	677 population	Descriptive study	Catalonia	tuberculosis, Chagas disease, hepatitis C, typhoid, scabies, hepatitis B, mumps, and tinea capitis.	Community-based Model	Identified the needs for Epidemiological surveillance of communicable diseases in migrant population. CB model facilitate access to health system even by migrants including undocumented migrants.	Strengthen Epidemiological surveillance system to reach all the cases and contacts.	Case management and contact tracing of migrants remain a challenge.

S023	[20]	Scopus	Digitalizing disease surveillance: experience from Sierra Leone	16 districts	Retrospective mixed method	Sierra Leone	Acute Flaccid Paralysis, Acute Viral Haemorrhagic fever, neonatal tetanus, yellow fever, measles, Poliovirus and anthrax, and other infectious diseases including dog and snake bites.	IDSR, District Health Information Software 2 DHIS2 and NMC	The eCBDS is an effective surveillance electronic tool used for capturing and submitting individual-level data on priority diseases in real-time and designed using the DHIS2 Tracker Module. The country had implemented the electronic case-based surveillance system across 95% of public health facilities in 16 districts. Improved quality of case-based surveillance data reported through eCBDS such as improved case notification, detection from health facilities.	Increase stakeholder engagement, awareness, fostering trust and confidence and buy-in of the system to ensure all key stakeholders are on board.	The development, setup, implementation, and maintenance of the electronic surveillance System is a challenge. Resources required to maintain an electronic system include stable internet infrastructure, usable electronic devices and data bundles or zero-rated internet access. The reporting nature of the case-based system requires several stages and different individuals. Therefore, delays in registering cases affect the capture of laboratory results. The stages in the flow of information are dependent on each other.
S024	[15]	Scopus	Epidemiologic surveillance for controlling the Covid-19 pandemic: types,	30 papers	Integrative Review	Documents from WHO, Euro-surveillance CDC, Saudi CDC, MOH and journals	Covid-19	IDSR system, epidemiologic surveillance	Types of Covid-19 surveillance includes routine surveillance (comprehensive, case-based, and aggregated weakly methods), active, wildlife, syndromic, sentinel and sentinel-syndromic methods.	Enhancing epidemiologic surveillance of Covid-19 is a crucial tool for rapid cases detection, containment of spread, enabling public health authorities to manage the risk of COVID-19,	The quality of surveillance in developing countries is constrained by resources and training. The main limitations of surveillance are under-ascertainment /under-reporting, lack

			challenges and implications			through search on PubMed& Medline.			Laboratory and hospital-based surveillance are another important types. Helplines, surveys, participatory electronic, digital and event-based surveillance are relatively new cost-effective methods.	allowing economic and social activity, and monitoring the longer-term trends and changing in the virus. Adoption of multiple and complementary surveillance systems can ensure broad coverage.	of timeliness and completeness of surveillance data.
S025	[54]	Scopus	Evaluation of the notifiable diseases surveillance system in South Africa	5 parameter estimates	Compa rative study	South Africa	Measles, meningo coccal meningiti s and typhoid.	Laboratory-based surveillance and Notifiable Disease Surveillance System	The study indicates that the laboratory system performed better than the NDSS in terms of completeness, stability and representativeness. The South African NDSS does not mandate zero reporting between levels of the healthcare system. 173 measles cases were positively identified by the NHLS but only 54 were notified. Meningococcal meningitis had 230 positive laboratory results with only 105 notifications received. Typhoid cases were 64 and 18 notified.	Both the clinicians and the laboratories should be required to notify regarding the diseases requiring notification, like in countries such as New Zealand, Ireland North Carolina) and Sweden) and some less developed countries such as Sri Lanka.	In SA only clinicians are required to notify the cases. Incorrect or incomplete recording of names,

S026	[14]	Scopus	Factors influencing operationalization of Integrated Disease Surveillance in Malawi	43 personnel	Case study design	Malawi: Lilongwe and Dowa districts	All notifiable diseases	Malawi Disease surveillance system, and IDSR system	There are numerous other vertical surveillance systems in other directorates in MoH and international donors. Inadequate funding leads to outdated digital equipment, limited access to electronic data transfer (internet, data bundles, and airtime), and insufficient server capacity for national-level data storage that also have specific data requirements. January and June 2022, IDSR program reported only 600,000 cases of malaria, whereas the malaria program had reported 2.4 million cases during the same period.	There is a need to improve operationalization of IDS in Malawi for it to fulfil its purpose as main national tool for surveillance and response. Strategies for enhancing IDS implementation could encompass review of the tools used, bolstering surveillance coordination and integration with vertical programs, coupled with providing robust support aligning with the IDSR framework.	Lack of integration of the systems leading to same data collected several times, like vaccine preventable diseases are both reported under EPI and under IDSR. Poor enforcement of reporting from private health facilities was perceived as a significant contributor to delayed or incomplete data. Lack of feedback among district and national managers. IDS frontline workers lacked training and technical guidelines. Case definitions were not available at point of reporting leading to confusion about what to report, such as whether to report suspected or confirmed cases of rabies or just dog bites,

S027	Donadel et al. [8]	2022	Web of Science	Comprehensive Vaccine-Preventable Disease Surveillance in the Western Pacific Region: A Literature Review on Integration of Surveillance Functions, 2000-2021	87 articles	Systematic Review	West-Pacific region	Measles, rubella, dengue, diarrheal diseases, rotaviruses, zika, Japanese encephalitis, diphtheria, pertussis	National integrated VPD surveillance systems – laboratory networks	Significant progress has been made toward the quality and sustainability of VPD surveillance systems in WPR countries, especially for diseases with eradication and elimination goals. 50% of WPR countries have included all or some VPDs in an integrated surveillance system. National integrated VPD surveillance systems with laboratory support exist for febrile rash illnesses, diarrheal diseases, arboviruses, and bacterial VPDs.	Any approach to integrated VPD surveillance must be flexible and able to respond to local conditions. Reported challenges in surveillance illustrate the need for improving efficiencies in resource utilization and strengthening integration of surveillance support functions at every level. Improving laboratory capacity through standardized test procedures, quality control, and integrated trainings is critical for successful implementation of an integrated system.	There is large variability of VPD surveillance maturity and performance across countries. In some WPR countries, VPD surveillance systems are parallel, duplicated, or fragmented. Several references noted fragmented systems and a lack of coordination between units as barriers to integration, highlighting the importance of engagement across public health units and between the public and private sectors. Noted the insufficient role of the private sector in disease surveillance.
S028	[47]		Scopus	Community-based surveillance of infectious diseases: a systematic review of drivers of success	19 sources (17 peer-reviewed and 2 grey literature) 1274 records published between 2012 and 2022.	Systematic Review	15 Countries	All diseases	Community-based surveillance	Included sources reported on community-based surveillance for the detection and reporting of a variety of diseases in 15 countries. The drivers of success were grouped based on factors relating to: (1)	The investment in participatory community engagement more broadly may be a key surveillance preparedness activity.	Strong supervision and training, a strong sense of responsibility for community health, effective engagement of community informants, proximity of surveillance workers to communities, the use of simple and

									surveillance workers, (2) the community, (3) case detection and reporting, (4) and integration.		adaptable case definitions, quality assurance, effective use of technology, and the use of data for real-time decision-making.
S029	[12]	Scopus	Lessons learned for surveillance system strengthening through capacity building and partnership engagement in post-Ebola Guinea, 2015–2019	4 objectives considered for strengthening surveillance system	Original research	Guinea	Ebola other priority diseases	Community-based surveillance, IDSR system, DHIS2	WHO facilitated the evaluation of Guinea's 2011 IDSR technical guidelines and developed recommendations for a simplified guide focused on the priority diseases and events identified by the Guinean government. For each priority disease and event, the generic case definitions and IDSR procedures from the 2011 guidelines were adapted for the Guinean context. The US CDC surveillance partners have helped strengthen early case detection by training more than 10,300 community health workers in 18 health districts to identify and report unusual health events and	Periodic evaluation to assess whether knowledge and proper application of IDSR tools is sustained. The government of Guinea should continue to use the GHS Index to monitor its performance overtime to identify risk factors and capacity gaps that can help inform technical strategies and financial commitments to establish sustained health security capacity	Among the indicators relevant to the surveillance strengthening activities described in this paper (zoonotic disease, real-time surveillance, and reporting, surveillance data accessibility and transparency, case-based investigation, and epidemiology workforce), no progress has been made from 2019 to 2021

									suspected cases of priority diseases from their community. The Ebola outbreak response in Guinea relied heavily on community volunteers, who could then be trained/re-trained to support other surveillance functions. Community health workers that supported other health activities before the Ebola outbreak provided a synergistic pool of personnel to support surveillance.		
S030	[41]	PubMed	Evaluation of two influenza disease surveillance systems in South Africa	205 sentinel sites with 18,293 SARI patients and 9,104 ILI patients	Comparative study	9 Provinces of South Africa	Influenza like illness and severe acute respiratory illness	Sentinel surveillance, ILI, and SARI system, hospital-based surveillance programme	Data quality was a major strength of the SARI system, with 40/43 key data elements on patient interview forms having completeness measures above 90% (n = 15,189 forms). Timeliness was a relative strength of the SARI programme, although there were weaknesses in terms of submission of case investigation forms.	To best monitor influenza in South Africa, we propose that both ILI and SARI should be under surveillance. Improving ILI surveillance will require better quality and more systematic data collection, and SARI surveillance should be expanded to be more nationally representative, even if this requires scaling	SARI system has high operating costs than ILI, hence its limited to few sentinel sites 4 in 9 provinces where the cape provinces are all excluded.

										back on information gathered.	
S031	[49]	2020	Scopus	Current knowledge of COVID-19 and infection prevention and control strategies in healthcare settings: A global analysis	7 guidelines	Review	2 high-income countries (Australia and United Kingdom) and 1 middle-income country (China)	Nosocomial Covid-19	Global Covid-19 IPC guidelines	4 guidelines recommend patient education, and 3 guidelines suggest establishing surveillance in the hospital to monitor cross infection in patients and HCPs. The transmission model and risk exposures of the COVID-19 pandemic were identified. The identified signs and symptoms of the case patients suggest that SARS-CoV-2 can be transmitted through cough, sneeze, saliva, nasal secretion, stool, and vomit via droplet, aerosol, faecal–oral, or faecal–droplet transmission.	The guidelines recommend that HCPs should wear surgical mask as a droplet precaution and during specimen collection. The use of N95 or equivalent respirators is recommended only during AGPs in all guidelines. Sufficient isolation space for patients	Currently discrepancies exist among the Guidelines as not all documents acknowledge the 3 routes of transmission. All the guidelines recommend early diagnosis and rapid isolation of COVID-19 patients though the studies have indicated that rapid diagnosis of patients is challenging since the signs and symptoms of COVID-19 are nonspecific and may be confused with all microbial causes of respiratory tract infection. Shortage of PPEs and isolation space in health facilities occurred.
S032	[43]	2020	PubMed	Diarrhoeal diseases in Soweto, South Africa, 2020: a cross-sectional community survey	374 households with 1640 individuals	Cross-sectional study	Soweto South Africa	Diarrheal diseases	Health and demographic surveillance site (HDSS)	Acute diarrhoea was reported to be the most common than the persistent diarrhoea. Of the 374 households surveyed, 78 (20.9%) reported at	Identified barriers were regarding accessing healthcare in Soweto, including issues with the health system (such	The current diarrheal cases records are based on healthcare-care level data as majority of diarrheal cases were not reported to the health facility

										least one diarrhoeal episode in the past 2 weeks. Seventy-one (91.0%) of these had a single episode per household, six (7.7%) had two episodes, and one (1.3%) had four episodes. Hence, a total of 87 diarrhoeal episodes were reported, 36 (41.4%) of which were self-reported by the respondent and 51 (58.6%) were reported on behalf of someone else in the household. The presence of children between 5 and 15 years in the household was significantly associated with episodes of diarrhoea. Since the diarrhoeal rate in this age group was similar to the rate for adults, it is likely that having a child of school-going age in the household is a risk factor for others in the household as these children may act as vectors	as deficiencies in healthcare delivery, dissatisfaction with services, medications being out of stock) and personal reasons (such as time, finance and transportation constraints)	

S033	[3]	Web of science	National Communicable Disease Surveillance System: A review on Information and Organizational Structures in Developed Countries	34 Studies	Review	Germany, US, Australia	All diseases	National Notifiable Disease Surveillance System, National Electronic Diseases Surveillance System,	In majority of countries the department of health (DoH) is responsible for managing this system. The reviewed countries use both NNDSS and NEDSS for disease surveillance. Electronic communicable diseases surveillance system can speed up the reporting process of diseases; facilitate data aggregation and managing bulks of data.	There is a need for improvement and upgrade of the current surveillance system relative to emerging infectious diseases. Furthermore, the experience and profiles of developed countries indicate that such systems could not be initiated so long as the proper infrastructures are not implemented for data exchanges across different centers.	Reporting of notifiable diseases is mandated only at state level. Poor communication among the centers and organization related to management of communicable diseases at different levels was one of the drawbacks detected in the studies countries along with shortage of human resources, underfunding, poor coordination among those involved in surveillance, and higher levels failing to give feedback to lower tiers.
S034	[65]	Web of Science	Challenges in Implementing Surveillance Tools of High-Income Countries (HICs) in Low Middle-Income Countries (LMICs)	3 countries	Review	India, Nepal, Sri-Lanka	All diseases. Dengue web-based surveillance,	IDSR system. a hospital-based sentinel surveillance, Health Management Information System,	The findings revealed that the Integrated Disease Surveillance and Response (IDSR) has been implemented successfully in most countries in Africa especially LMICs. IDSR was implemented in 44 LMICs by December 2017, and 70% had electronic IDSR systems. However,	Although challenges were noted regarding the insufficient resources and integration of health systems. Integrated Surveillance Systems should be implemented in all countries using modern technology to improve the speed of detection and control of	Some infections do not have proper diagnostic facilities. An important capacity limitation in clinical laboratories of LMICs is identification of antimicrobial resistant organisms as well as other pathogens to species level. This affects the surveillance of infections and

									the target of at least 90% IDSR implementation coverage at the peripheral level was achieved only by 12 countries.	outbreaks and to implement infection prevention and control measures in community setting. Capacity building in healthcare with adequate number of trained healthcare workers and infection preventionists will be important to improve the surveillance of healthcare associated infections. Improving laboratory capacity with proper quality assurance programmes is important to support communicable disease surveillance in the community as well as for surveillance of HAI and AMR. Data communication methods among all types and levels of healthcare institutions and the national and regional surveillance centres should be improved in LMICs.	antimicrobial resistance. the integrated surveillance of communicable diseases in HICs is not happening in timely manner, and GPS and other novel technology are not available. Less information is available on Health facility Acquired Infections data from Africa. In South Africa, inadequate human resources and manual data entry were two main reasons given for poor HAI surveillance.

S035	[51]		Web of Science	Engaging with uncertainty: Information practices in the context of disease surveillance in Burkina Faso	1266 suspected cases	Practice perspective= quantitative tools	Burkina Faso	Dengue outbreak	Health Information System, DHIS2,IDSR	The empirical research identified conditions that contributed to uncertainty in the context of disease surveillance and response in a LMIC setting. Including how people deal with it and the role that HIS played in helping to respond to it. Uncertainty served as an analytical tool to help foreground the conditions that shaped what the actors did not know and urges the analysis of alternative ways of acting beyond the formal and routine. Among adults, the biggest burden of disease are lower respiratory infections and malaria.	Protocols are powerful in guiding practice, producing certainty by their very formal outlining of responsibilities, of what to do, how, when, and by whom, providing a sense of ontological security.	Lack of reporting was, in some cases, due to the limitations of the protocol, which only focused on the thirteen selected diseases.
S036	[4]	2010	PubMed	A niche for infectious disease in environmental health: rethinking the toxicological paradigm	National Science Foundation, the National Institutes of Health, and the National Institute of	Literature review	US	Infectious disease Surveillance	Environmental exposures research, Population based research to determine the association between	Environmental health encompasses complex disease processes, many of which involve interactions among multiple risk factors, including toxicant exposures, pathogens, and susceptibility.	Research initiatives should be designed to foster collaborations among researchers in infectious disease and environmental health, specifically toxicology. Large, population-based studies should	The toxicological paradigm has not been engaged previously as a mechanism for understanding the relationship between environmental health and infectious diseases.

					Environmental Health Sciences.				environmental exposures and the occurrence of infectious diseases.	Reframing the toxicological paradigm provides a roadmap for interdisciplinary research and a guide for research conducted at this important interface, and may help public health practitioners to weigh the risks posed by the constant barrage of environmental exposures on the human system and determine how to best mitigate them. Some differences between the toxicological paradigm and the infectious disease model challenge their integration. Although some epidemiological studies in environmental health focus on populations [largely with ecological designs, e.g., those in air pollution research , the classic toxicological paradigm	include sample collections that enable assessment of exposures to environmental agents as well as to pathogens. Funding and program mandates for environmental health studies should be expanded to include pathogens to capture the true scope of these overlapping risks, thus creating more effective research investments with greater relevance to the complexity of real-world exposures and multifactorial health outcomes. A new model that integrates the toxicology and infectious disease paradigms should be developed to facilitate improved collaboration and communication by providing a framework for interdisciplinary research. Pathogens should be part of environmental health research planning	Basic science research funding focuses on laboratory-based inquiry. more than on disease processes in the context of the environment or large populations. No projects funded by the EID studied interactions between toxicant exposures and infectious disease.<br><br>Public Health Service agencies concerned with the environment, such as the National Institute of Environmental Health Sciences (NIEHS), research has not emphasized infectious disease.

										models of toxicokinetic and toxicodynamic at the level of the individual, generalizing from this level to a similarly exposed population. In contrast, models of infection are based on population-level probabilities of progressing from exposure to infected, infectious, or immune/ susceptible states. The concept of "risk transmission" is critical to infectious Figure 1. Integrated toxicological–pathogen conceptual paradigm for disease etiology. Disease dynamics.	and funding allocation, as well as applications such as surveillance and policy development.	
S037	[5]	2011	PubMed	The case of cholera preparedness, response and prevention in the SADC region: A need for proactive and multi-level communicati	10 SADC countries: Zimbabwe, Zambia, Swaziland, South Africa, Namibia, Mozambique, Malawi, Botswana, Angola	Qualitative mixed method data collection	SADC regions	Cholera	Water testing, Develop an early warning system for prediction of Cholera outbreaks caused by environmental change.	A few SADC countries effectively monitor and manage groundwater use sustainably. In the absence of effective monitoring and surveillance systems and streamlined reporting procedures, little can be done to curb the contamination of groundwater that	The need to recognize the outbreak, rapidly mobilize resources to the affected area, dispense antibiotics or vaccines to the population, and follow up with patients to confirm that the intervention has been appropriate and effective is crucial.	In many developing countries, the approach to cholera outbreaks is a reactive 'emergency response,' as many are unprepared when the outbreak occurs, and actions are directed at controlling the outbreak and minimizing mortality. Cholera thrives in environments where

				on and co-ordination						exposes millions of people living in rural areas to waterborne diseases. The provision of rural water supply has improved considerably over the last decade, with access to improved water sources increasing from 56% in 1990 to 64% in 2006 in Africa (WHO, 2008). However, in some countries, such as Zimbabwe and Zambia, urban water services coverage has decreased	Administering mass vaccinations. alone, however, will not prevent or control the spread of cholera. Policymakers also need to be mindful of how poor infrastructure and health services may impede the efficacy of these vaccinations. In addition, financial resources need to be provided for surveillance, education, and additional medical supplies. Long term: • Ensuring that all people in the country have provision for safe water, sanitation, hygiene, and health services. Areas that are known to be prone to cholera outbreaks should be given priority. • Ongoing education of health workers as well as communities to help with the prevention and management of future outbreaks. Strengthened monitoring and	there is poor infrastructural development, particularly in terms of access to running water, sanitation, and healthcare services. Many challenges, including budget constraints, lack of healthcare materials, poor maintenance and operation of water infrastructure, and weak early warning systems in many countries in the SADC region. The issue of who is responsible for responding to cholera outbreaks is a 'grey area' in an ever more interconnected and globalised world.

											surveillance of environmental data as well as disease data to help with early detection and control of cholera outbreaks.	
S038	[9]	2013	Google Scholar	Rodent control in urban communities in Johannesburg, South Africa: from research to action	Five residential areas in the City of Johannesburg	Longitudinal panel study	South Africa-City of Johannesburg	Vector-borne disease	Rodent infestation monitoring and control, Waste management, and investigating rodent-borne diseases. Health education.	This analysis revealed that rodents are perceived to be a significant problem in urban settlements in Johannesburg, particularly in the relatively impoverished areas of Hospital Hill, Bertrams, and Braamfischerville. The reported rodent infestation rates of 26.5–69.0% in this study are as high or higher than those reported in other studies. The City of Johannesburg has expressed a commitment to conduct "regular 'blitzes' throughout the City to determine the extent of rodent infestations with the view to instituting the necessary remedial measures".	Municipal managers must urgently address issues of education and the provision of water, toilets, and refuse disposal services in communities without adequate access to these services. It is encouraging to note that the City of Johannesburg has expressed a commitment to conduct "regular 'blitzes' throughout the City to determine the extent of rodent infestations with the view to instituting the necessary remedial measures". Community participation should be emphasised as it is also essential to achieve rodent control.	Several departments share the responsibility for rodent control, including the water and sewerage authorities, the public works department, the city housing authority, the parks and recreation departments, as well as the public health department. A particular challenge is the integration of city departments in a comprehensive rodent control programme, involving inter-sectoral planning and coordination. While this study demonstrated the need for an integrated, sustainable rodent control programme, it is often found that what is learned in research settings is

												not often translated into practice.
S039	[10]	2012	Web of Science	Epidemiological investigation into the introduction and factors for the spread of Peste des Petits Ruminants, southern Tanzania: Proceedings	Eight villages	Cross-sectional study	Tanzania	All disease	Community-based surveillance: Contact tracing, Inspection of animal keeping facilities. awareness Regular health inspections are conducted in collaboration with veterinary workers. Samples collection for Competitive Enzyme Linked Immunosorbent Assay Analysis.	The findings have confirmed the introduction of PPR in the south for the first time. Tanzania. The presence of PPR poses a high risk of the disease spreading southward to other areas in southern African countries within the SADC region, thus necessitating concerted and collaborative efforts to prevent and control it while averting losses. It was discovered that the source of the disease was the introduction of new animals purchased from the live animal market. Similar sources of the disease have been implicated before	Engaging community-based environmental health practitioners and recognising them as experts in their communities is vital to ensure the successful planning, development, implementation, and evaluation of the EHCR process	Delayed confirmation, as it took only one year from the first official confirmation of the disease in northern Tanzania to introduce it in southern Tanzania, it has taken about two years to confirm this disease through the efforts of the current study

S040	[16]	2013	PubMed	Environmental health in South Africa	Not applicable	Narrative review	South Africa	Environmentally induced communicable diseases	Surveillance of premises, the burden of ill health is attributable to environmental factors. Addressing ecological hazards in the places in which people live, learn, and play. Water quality, food safety, vector control, waste management, disposal of the dead, surveillance and prevention of communicable diseases, air quality monitoring	History provides strong evidence for the decisive role that environmental health action and professionals can play in preventing disease and promoting health. South Africa grapples with multiple, simultaneous burdens of disease: Environmental factors play a role in the causation and prevention of each of these. Five environmental risk factors (unsafe water, sanitation, and hygiene; indoor air pollution from household use of solid fuels; urban outdoor air pollution, and lead exposure) were associated with 5% of all deaths in South Africa in 2000. The joint attributable burden was exceptionally high in children under five years of age, accounting for nearly 11% of total deaths in this age group.	Tackling the large-scale and complex environmental health problems in South Africa will require the pooled expertise and experience of multiple disciplines and sectors, including non-government organisations, the media, and public pressure groups.	Several steps have been taken to enhance environmental health in South Africa, ranging from legislative frameworks to community engagement. Though it still faces numerous formidable and complex environmental health challenges. These include problems emanating from improper mining, agricultural and industrial practices, and under- or inappropriately developed human settlements. With the advent of climate change and increased climate variability, there is growing concern that existing environmental health challenges are likely to intensify.<br>South African environmental health services appear to be stagnant or in decline. In part, this decline is attributable to unresolved issues arising from a

												protracted and incomplete transformation process, such as unfunded or inadequately funded environmental health mandates (particularly at the district level), an inadequate EHP: population ratio, and a high degree of inequality in environmental health service provision across the country.
S041	[21]	2013	Google Scholar	Integration of Water, Sanitation, and Hygiene for the Prevention and Control of Neglected Tropical Diseases: A Rationale for Inter-Sectoral Collaboration	Four Key collaboration areas: (i) advocacy, policy, and communication; (ii) capacity building and training; (iii) mapping, data collection, and monitoring; and (iv) research. Discussed by	Systematic review	Global study	Neglected tropical diseases	Ensure adequate and equitable access to water and sanitation, and practice good hygiene. Collaborate with stakeholders for control and prevention of NTDs	A systematic review and meta-analysis established a correlation between soil-transmitted helminth infections and sanitation. Access to, and use of, sanitary facilities was correlated with lower odds of soil-transmitted helminth infection, including 0.54 (95% confidence interval [CI] 0.43–0.69) for Ascaris lumbricoides, 0.58 (95% CI 0.45–0.75) for Trichuris trichiura, and 0.60 (95% CI 0.48–0.75) for hookworm [21].	Persistent challenges to collaboration between the WASH and NTD sectors must be acknowledged and confronted. To overcome barriers, the benefits of collaboration for each sector need to be clearly established and articulated, and common goals institutionalized in global and national policies and progress indicators. Sector-wide coordination and program integration may not be feasible ways	Key stumbling blocks for collaboration include differences in the scale of interventions, indefinite timelines for WASH investments, and community engagement on one hand, and a large disparity between costs of WASH services in comparison to a primarily treatment-based control approach

					researchers , practitioners, donors, and representatives from advocacy, relief, and development NGOs.					However, no randomized controlled trials were found, and the strength of the available data was limited. There are currently no similar reviews assessing the correlation between soil-transmitted helminthiasis and access to water or hygiene. Two Cochrane reviews revealed only very few experimental studies assessing face washing	forward within the current policy climate and existing funding structures, but strategic areas for sectoral and partner-level coordination, collaboration, and communication have been identified	
S042	[22]	2012	PubMed	Infectious diseases following natural disasters: prevention and control measures	21 articles	Review	(Bangladesh, Iran, Indonesia, Brazil, Mozambique, Pakistan, Thailand, India, Haiti, other affected African countries	Malaria, and other disaster related diseases	Diseases surveillance and disease risk assessment, Shelter inspection and water and sanitation services. Solid waste management , Community outreach/ health education on hygiene,	The prolonged health impact of natural disasters on a community may lead to the collapse of health facilities and healthcare systems, disruptions in surveillance and health programs (such as immunization and vector control programs), limitations or destruction of farming activities (resulting in food scarcity or food insecurity), and	Most of the publications on disasters and communicable diseases are limited to morbidity and mortality studies. Therefore, there should be further studies in areas such as the socioeconomic burden of disasters and subsequent infectious diseases, and the potential of information communication technology (e.g., mobile health and e-learning) in disaster	No documentation on geomorphologic disaster (e.g., avalanche and landslide) associated with infectious disease transmissions and outbreaks was found.

										interruptions in ongoing treatments along with the use of unprescribed medications. Increases in infectious disease transmission and outbreaks following natural disasters are linked to the prolonged after-effects of the disaster. The public health consequences of flooding include disease outbreaks resulting from the displacement of people into overcrowded camps and cross-contamination of water sources with faecal material and toxic chemicals. Flooding is often followed by the proliferation of mosquitoes, leading to an increase in mosquito-borne diseases, such as malaria. Earthquake disasters are the second most reported natural disaster (after floods) and the most prevalent among geophysical disasters.	management and disease prevention in developing countries. Infectious disease outbreaks result secondarily from the exacerbation of disease risk factors. Therefore, the rapid implementation of control measures should be a priority in communities displaced by disasters, especially in the absence of pre-disaster surveillance data. Surveillance in areas affected by disasters, in camps, health facilities (hospitals and clinics), and other locations where victims are treated, is crucial. It is also essential to consider stepping up the existing system (e.g., national surveillance of reportable diseases). Management protocols should be established per national guidelines. Surveillance and early warning systems should	

											include diseases that are already endemic as well as unusual events that could occur, since new pathogens can be imported or can emerge and re-emerge in the disaster-affected region. To increase the ability to control infectious diseases and prevent epidemics following disasters, preparedness measures must be taken before disasters occur.<br>In disaster situations, education on hygiene and hand washing, provision of adequate quantities of safe water, sanitation facilities and appropriate shelter are very important for the prevention of infectious diseases	
S043	[23]	2015	Web of Science	Cleaning and disinfecting environmental surfaces in health care: Toward an integrated	40 members from 4 countries	NORA framework. Survey	United States	Nosocomial infections	Environmental services workers are the leading occupational group performing cleaning and	Health care infection prevention and occupational health are often practiced separately.	There is a need to develop a more integrated approach that minimizes harmful exposures to cleaning and disinfecting for healthcare workers	However, extensive observational studies show overall that the thoroughness of manual terminal cleaning is currently lacking. Health care infection prevention

				framework for infection and occupational illness prevention					disinfecting activities in health care facilities: environmental services directors and managers oversee their work.		and patients, without compromising the effectiveness of infection prevention efforts. There is a need to understand the effectiveness of better cleaning and disinfecting products and procedures to reduce the incidence of infectious diseases and colonization in health care workers and patients. There is a need to evaluate the extent to which contact with surfaces that are contaminated with infectious agents contribute to HAIs in patients and to occupationally acquired infections in health care workers, as well as to evaluate the potential of environmental surfaces to transmit infections to health care workers and patients in health care settings other than hospitals (eg, nursing homes, ambulatory	and occupational health are often practiced separately.

											care settings, home health care).	
S044	[24]	2015	PubMed	The 2008/2009 cholera outbreak in Harare, Zimbabwe: case of failure in urban environmental health and planning.	Key stakeholders	Commentary review	Zimbabwe	Cholera	Environmental surveillance, and Health promotion services	The paper highlights the inadequacy of safe and clean water in most of the suburbs, the collapse of the waste management, water supply and sanitation systems of the city as the major explanations for the scourge.	The paper concludes that sustainable urban health initiatives involving both central and local government will provide a long-term solution to the problems highlighted.	Harare remains troubled with the shortage of purification chemicals, making it quite impossible to supply clean and adequate water. The national economic crisis also had a strong bearing against the performance of the public health sector.
S045	[25]	2015	PubMed	Environmental and Behavioural Determinants of Leptospirosis Transmission: A Systematic Review	2723 selected and 53 fulfilled criteria	Systematic Review	Asia.	Leptospirosis	Water and sanitation monitoring	The review confirms the complex environmental transmission pathways of leptospirosis, as previously established. Although floods emerged as some of the most critical drivers on islands and in Asia, the consistent pattern observed regarding exposure to rodents and behavioral and sanitation-related risk factors indicates potential areas for	A better understanding is crucial for the planning and implementation of effective interventions. Future attempts to develop leptospirosis transmission models should primarily address environmental water related exposures as a main driver for transmission. Future epidemiological studies should	The information on leptospirosis in Africa is scarce, we included all identified studies from this continent. However, only three eligible studies were conducted in Africa. A study in Ghana assessed agriculture (cocoa farming) and from the two conducted in Nigeria one considered the risk of disease in abattoir workers and one in kennel

										intervention. Water-associated exposures were, with few exceptions, linked to an increased risk of leptospirosis. In resource-poor countries, floods and rainfall held particular significance, while recreational water activities were more relevant in developed countries. Rodents were associated with a heightened risk of leptospirosis, but the variation among studies was considerable, likely partly attributable to differences in exposure definitions. Livestock contact was generally linked to increased risk; however, several studies found no association. The median odds ratios associated with contact with dogs and cats were close to unity. Sanitation and behavioral risk factors were almost always strongly linked to	address ecological, climatic and rodent demographic components for a more detailed understanding of environmental contamination.	workers. In all cases, the investigated activities were seen to increase risk of leptospirosis transmission.

										leptospirosis, although their impact was rarely quantified. investigated		
S046	[50]		Scopus	A One Health Evaluation of the Southern African Centre for Infectious Disease Surveillance	Five countries:	Mixed method approach	Tanzania, Zambia, Mozambique, DRC, and South Africa	Infectious diseases	Disease surveillance, Infectious disease investigation, Health education, trace contacts, and environmental risk assessment	SACIDS’ strategy is concentrated on enhancing the capacity of institutions for the detection, identification, and monitoring of infectious diseases. By promoting joint efforts in education, communication, research, and disease surveillance, SACIDS is a pioneer initiative in the adoption and application of OH principles for the surveillance of infectious diseases in Southern Africa. there is currently a disconnect between the rationale and motivation for SACIDS and the actual outcomes and impact. Consequently, there is no assessment of improvements in attributes that define infectious disease surveillance, such as performance or	All factors call for a syndemic approach to tackle infectious diseases through better collaboration between human health, animal health, and environmental health, and socioeconomic sectors	The synergy of the health and economic consequences of such diseases, amplified by the poor governance systems and civil instabilities, the lack of participatory health policies, of personnel and of resources and the need for adequate leadership, exacerbates poverty. .

									functional attributes of surveillance or their OH integration. To facilitate future program success and achievement of outcomes and impact, it is recommended that SACIDS develops measurable indicators in line with their theory of change and implements relevant data collection and evaluation activities		
S047	[36]	2018	PubMed	Active Case Finding for Communicable Diseases in Prison Settings: Increasing Testing Coverage and Uptake Among the Prison Population in the European Union/European Economic Area	A total of 7,041 were retrieved, but only 14 records reported findings from the EU/EEA region	Systematic Review	only literature from EU/EEA countries, EU candidates countries (i.e., Albania, Bosnia-Herzegovina, Montenegro, Serbia, Turkey), and other Westernized countries (i.e., Australia,	TB. STIs, BBVs	Investigation of notifiable diseases. Disease health education, Prison Facility inspection.	The search highlighted some important gaps and limitations of the existing evidence. "Most of the existing evidence on active case finding in prison settings are concentrated on a few communicable diseases, namely BBVs, STIs and TB. These findings may be consistent with the general notion that these diseases constitute a sizeable disease burden in the prison population, and [that there is] higher risk of	Due to limited evidence on active case finding in prisons within the EU/EEA, with no studies providing statistically significant evidence of the clear benefit of any single approach over others. As a result, it is challenging to conclude the effect of different testing approaches, and more comparative studies would be needed to assess the effectiveness and influence of different active case finding	Our search retrieved a limited number of studies that provided a comparative analysis of different active case finding methods. Notification of testing results was seldom reported, with the notable exception of HIV testing. Conversely, treatment initiation was frequently described for STIs and TB, with important variations across countries. Linkage to care after release was hardly reported at all; thus information essential

							Canada, New Zealand, Switzerland, and the United States).			transmission within prison settings	strategies in correctional facilities of the EU/EEA.	for assessing the medium- to long-term outcomes of active case finding activities in prison settings is not provided.<br>Others were unable to be retrieve any evidence on testing for several communicable diseases (e.g., parasitic diseases) in correctional facilities.
S048	[38]	2018	PubMed	Comparing the behavioural impact of a nudge-based handwashing intervention to high-intensity hygiene education: a cluster-randomised trial in rural Bangladesh	450 students in 20 schools	Cluster-randomised trial	Bangladesh	None	Health and hygiene education. Water and sanitation monitoring. Infrastructure/ school inspection	The trial demonstrated sustained improved handwashing behaviour 5 months after the nudge intervention. The nudge intervention's comparable performance to a high-intensity hygiene education intervention is encouraging. The nudge intervention and the HE intervention were found to be equally effective in sustaining their impact over 5 months post-intervention (adjusted IRR 0.81, 95% CI 0.61–1.09). When	The use of nudges to promote HWWS is still in the early stages of development, the findings of this RCT call for further and more robust studies of the nudge-based interventions.	

										comparing intervention delivery timing, the simultaneous delivery of the Hygiene Education intervention significantly out-performed the sequential HE delivery (adjusted IRR 1.58 CI 1.20–2.08), whereas no significant difference was observed between sequential and simultaneous nudge intervention delivery (adjusted IRR 0.75, 95% CI 0.48–1.17).		
S049	[62]	2019	Google Scholar	Outbreaks and Epidemics of Malaria in the SADC Region. Epidemics and the Health of African Nations	16 Regions	Review Report	SADC regions: Tanzania, South Africa, Malawi, Zimbabwe, Zambia, Angola, MozambiqueMadagascar, Namibia, Eswatini, Botswana, DRC	Malaria	IDSR, DHIS2, Vector control (monitoring indoor residual spraying services). Investigate malaria notifications	There was an unexpected upsurge in the number of cases and deaths after significant gains in the preceding period. In countries with moderate to high transmission rates such as those in Malawi, Mozambique, DRC, Angola and Madagascar had a high incidence of malaria, whereas Tanzania has a low	To prevent outbreaks or epidemics, countries need to ensure that All communities receive health education aimed at behaviour change. This should go hand in hand with ensuring universal access to all interventions. Behaviour change should be primarily related to climatic conditions conducive	Weakening of the malaria control programme, caused by funding. shortages, complacency, poor strategy execution, and cessation of control activities; • increases in the intrinsic potential for malaria transmission – due to movement of humans or mosquitoes, development, land-use changes, and

										incidence, with high mortalities in Angola and the DRC. In countries with low transmission rates, such as South Africa, Botswana, and Eswatini, the incidence and mortality rates remain low.	to malaria transmission, to prepare the population to take precautions. Countries also need to establish strong malaria surveillance systems, as effectively countering malaria relies on accuracy and complete identification of and reaction to outbreaks.	changes in climate and weather patterns; and • technical problems, including drug and insecticide resistance, as manifested in vector and drug resistance.
S050	[45]	2019	Google Scholar	Co-developing climate services for public health: Stakeholder needs and perceptions for the prevention and control of Aedes-transmitted diseases in the Caribbean	73 stakeholders	Survey-Cross-sectional study	Dominica and Barbados.	Arboviral diseases	Surveillance. (Investigation of notified cases) Vector control. Awareness. The EHD has a mandate in nine program areas, including vector control, food safety, occupational health, port health, school health, and institutional hygiene.	Health practitioners indicated that their jurisdiction is currently experiencing an increased risk of arboviral diseases associated with climate variability, and most anticipate that this risk will continue to grow in the future. National health sectors reported financial limitations and a lack of technical expertise in geographic information systems (GIS), statistics, and modelling, which constrained the implementation of climate services for arboviruses—a	Establishing climate services for health as a mandate in the NMHS and health sectors is key to allow working in interdisciplinary teams. Beyond the climate and health sectors, a complex web of institutional actors who can engage strategically in the development of climate services for health should be identified. This finding highlights the importance of intersectoral collaborations, a key element of Integrated Vector Management for arbovirus control.	Insecticide resistance in Ae. aegypti is a major challenge for the health sector, given their reliance on chemical control to reduce disease transmission and lack of regulation of private vector control companies in Barbados. Community mobilization for arbovirus control is an ongoing challenge throughout dengue-endemic regions, requiring a nuanced understanding of local community perceptions and behaviour. key difference is that the study focused on

										shortage of personnel. The importance of strengthening partnerships with the private sector, academia, and civil society, and identified a gap in local research on climate-arbovirus linkages. Strategies to strengthen the climate-health partnership included a top-down approach, engaging senior leadership, multi-lateral collaboration agreements, national committees on climate and health. Mechanisms for mainstreaming climate services into health operations to control arboviruses included the development of climatic health bulletins and an online GIS platform. A 3-month forecast of arboviral illness for an epidemic forecast.	Health stakeholders stressed the need to increase analytical capabilities, such as GIS skills. User-friendly analytic tools/ instruments that combine health and climate information could be developed for the health sector for use in routine reporting activities. Creating National Adaptation Plans for Health can include climate considerations in health sector decision-making, providing an opportunity to strengthen climate services.<br>To engage senior leadership in the establishment of collaboration agreements (MOUs) between the climate and health sectors.	people working with arboviruses, environmental health, and climate, whereas other studies focused on health-care providers or public health professionals in general.
S051	[48]	2019	PubMe d	The Critical Role of Compliance in Delivering Health Gains from	N/A	Perspec tive paper	Georgia	None	WASH studies, Microbial risk assessment,	Compliance can vary by group. Some participants may report high compliance for normative practices,	Advocate for future studies that measure both key compliance behaviours and incorporate direct	Most environmental health intervention trials are “technology-led” and underemphasize the importance of

				Environmental Health Interventions					Exposure assessment,	especially those that convey perceived social status (e.g., cleanliness). Others may not comply with a given technology because of cultural, societal, and/or economic factors. Compliance can also vary by time; for example, intervention compliance may be more likely when risk is high due to heightened awareness Hand hygiene and influenza is a good example of this phenomenon.	measures of exposure, providing for a fuller assessment of the causal pathway beyond simple exposure to the technology and delivery of a distal health outcome. Although studies of this type are rare in environmental health.	human behaviours including compliance as integral to reducing exposures and, therefore, realizing health effects of interventions. Compliance can rarely be assumed or taken for granted, and doing so distorts our view of the evidence by ignoring this critical link between technology access and exposure.
S052	[53]	2021	Scopus	Management of environmental health to prevent an outbreak of COVID-19: a review	Global	Review Chapter	Global literature	COVID-19,	Management of COVID-19-contaminated waste/ infectious waste. Contact tracing. Ensure compliance with environmental health measures.	Different types of medical and hazardous wastes, such as used personal protective equipment and laboratory waste, were generated during the outbreak, along with a significant volume of non-infected items of the same nature. All of these must be identified and segregated before decontamination	Recommendations from the WHO and the Environmental Protection Agency (EPA) indicate that the risk of SARS-CoV-2 in drinking water is very low and encourage continued using drinking tap water. However, this concern remains unknown in countries with inadequate water supply systems that are not equipped	Valuable studies have been conducted on tracking SARS-CoV-2 in various water and wastewater systems around the world. However, many countries, due to a lack of facilities and equipment in this field, have been unable to monitor wastewater-based epidemiology. This method is one of the low-cost ways to

									Air quality monitoring	and/or disposal. Sound and safe management of this biomedical and health care waste, in parallel with safe routine procedures, is essential to prevent the dispersal of biological agents and control the risk of infection. Such effective management of this waste includes disinfection procedures, personnel protection, and training. several measures are recommended to reduce airborne contamination risk, such as avoiding crowded communities, immediate tracing, and diagnosis of asymptomatic bearers for quarantine or treatment, and the use of face masks in crowded places	to eradicate viruses, so it requires further investigation. Because the SARS-CoV-2 virus cannot easily overwhelm the advanced stages of water treatment, including filtration and disinfection. Since the present studies are unable to confirm the effect of climate conditions on the spread of COVID-19, further studies are needed to investigate the impact of temperature, humidity, and other factors on this phenomenon. More research should be conducted on municipal and medical waste management during the COVID-19 outbreak and its short- and long-term effects.	detect and control COVID-19 by preventing subsequent peaks, so it requires special attention in poorer and developing countries.<br>The first appearance of a new version of this virus, called COVID-20, derived from the UK and rapidly spread to many countries, puts a heavy burden on researchers to accelerate their intensive studies to find a global vaccine that can attach to all COVID viruses.
S053	[55]	2021	Google Scholar	The role of Environmental Health in preventing antimicrobial resistance in low- and	Two countries	Commentary	Uganda and Malawi	Diarrhoeal diseases	EHPs roles in reducing the spread of AMR include water sampling	EHPs have a key role in preventing the occurrence of AMR, including through health education and promotion, surveillance,	Understanding environmental pathways of AMR is vital for health professionals and communities to interrupt the	Lack of surveillance and up-to-date information regarding AMR among LMIC populations for the

				middle-income countries					and analysis, Sanitary inspection of water sources, Inspection of premises, including schools, markets, landing sites, and other Institutions, Inspection of food, abattoirs and public eating places, Medical examination of food handlers, Health education and promotion, Surveillance, Enforcement of legislation and Research.	enforcement of legislation, and research. The key Environmental Health practices in the prevention of AMR include: (1) adequate WASH through access to safe water; suitable containment, treatment and disposal of human excreta. Good personal hygiene practices to prevent the spread of resistant microorganisms, and contraction of illnesses which may require antimicrobial treatment; (2) proper disposal of solid waste, including the disposal of unused and expired antimicrobials; and (3) ensuring proper food hygiene and safety practices.	development and spread of resistant organisms, including ingestion of antimicrobial residues. Tis commentary describes key Environmental Health factors and the role of the discipline in the prevention of AMR in LMICs. Acceleration of WASH interventions (both infrastructure and behaviours) including proper handwashing with soap, clean play environments for children, animal containment, and clean water are critical for reducing both diarrhoeal diseases and the distribution of resistant organisms and genes.	prevention and treatment of resistant infections. Lack of sufficient sanitary facilities such as latrines and basic hygiene practices, particularly handwashing in LMICs, can lead to the spread of resistant organisms in the environment. Lack of collaboration with all health professionals dealing with infectious diseases.
S054	[52]	2022	Google Scholar	Local Government Environmental Health Services: Fundamentals for Effective	112 Sources Municipalities of South Africa	Grounded theory study – Integrated Literat	South Africa	None	Environmental risk management . water quality monitoring; waste management	The basis of effectiveness, according to the literature, is setting well-defined goals and directives in a mandate that clarifies levels of authority	Effective service delivery, therefore, is the responsibility, obligation, and professional commitment of both the institution and of the functionaries	EHS functions are intertwined with other BMS functions responsible for providing, operating, and maintaining infrastructure and services. To

				Municipal Service Delivery and Preventive Health Outcomes		ure Review			; environmental pollution control.	and functions. The mandate in the public sphere flows downstream from the highest authority, the constitution, to national, provincial, and local authorities and stems from the government’s policy framework and international accords, e.g., the United Nations Sustainable Development Goals (SDGs) and the WHO's Health in All Policies (HiAPs) initiative. About MHS and BMS, it is described in 14 sections and two schedules of the constitution and further outlined in acts of parliament and regulations such as the Municipal Structures Act (1998) and the National Health Act (2003). A mandate has two main components: i) the scope of the functions or that which an organisation, department, or	personally. And the numerous structural and functional intersections and overlaps require a holistic and integrated approach to ensure co-ordination, collaboration, and communication. This requires planning by synchronising objectives and key performance indicators (KPIs), aligning programming or scheduling of tasks, coordinated control, and ensuring adequate and appropriate resources to execute the mandate.	effectively fulfil its task, it necessitates integrating various and diverse administrative and managerial functions.

										individual is obliged to do and achieve; and ii) the scope of the authority or the level of control they have been allocated to execute the functions.		
S055	[56]	2022	PubMed	The first WHO global survey on infection prevention and control in health-care facilities	81 countries, 4440 responses	Cross-sectional study	Global study-across all six WHO regions (Africa, Americas, Eastern Mediterranean, Europe, South-East Asia, and the Western Pacific)	Nosocomial infections	Collaborate with IPC for hand hygiene programs, WASH, Water, and sanitation—infectious waste management. Health facility monitoring	Previous study results varied by income level. Although median scores in Austria and Germany reflected advanced levels of performance, those in Ghana and Pakistan reflected levels ranging from intermediate to basic or inadequate. Additionally, a national study in Georgia used an adapted version of the IPCAF; a systematic review of studies in mainland China described the assessment of IPC implementation according to the WHO core components; and a study in Kenya assessed facilities using the water, sanitation, and hygiene facility	Efforts to support the long-term development of IPC programmes and stepwise improvement are crucial, particularly in low-income and middle-income countries, which remain the most vulnerable. The findings show that further investments are needed in all countries to improve the effective implementation of IPC training programmes and meet adequate workload and staffing requirements, as well as standards for bed occupancy and spacing between beds. In low-resource settings, efforts are particularly needed to improve HAI	Gaps in IPC implementation, and key opportunities for improvement to inform ongoing global IPC improvement efforts, particularly in low-income and lower middle-income countries, which showed significantly lower IPC implementation. Past and present epidemics have shown how rapidly a few cases of infection by an emerging pathogen in a health-care facility can become a large outbreak due to poor IPC implementation. The endemic burden of HAIs and AMR continues to affect patient safety, hamper high standards of quality of care, and impede

										improvement tool. Most other identified studies focused on hand hygiene programme implementation, as well as needs, access, and availability of individual IPC elements at the country level. Overall, health-care facilities had an advanced level of IPC implementation, but this varied across income levels. Although most facilities reported having an IPC programme, few met all IPC minimum requirements recommended by WHO.	surveillance and IPC monitoring and feedback. Although some of these investments might have been made after this survey to combat the COVID-19 pandemic, ensuring sustained long-term implementation through stronger IPC policies and regulations, a competent and adequate workforce, and leadership support at the highest levels through dedicated budgets and accountability mechanisms is crucial	the achievement of universal health coverage.
S056	[59]	2024	PubMed	Assessment of SADC Countries' National Adaptation Planning Health Impacts Inclusion: A Thorough Review	Five SADC countries—Botswana, Mozambique, Namibia, South Africa, and Zimbabwe.	Review	SADC regions	Water, food, and vector-borne disease	Community health education and the investigation of waterborne, foodborne, and vector-borne diseases have emerged because of	While all five countries prioritized health, only South Africa had completed a National Climate Change and Health Adaptation Plan by 2021. Botswana, Mozambique, Namibia, and Zimbabwe reported that H-NAPs were in progress, but no additional	National strategies and implementation programs in SADC countries must be agile in their ability to scale and adapt while incorporating measurable actions and clear timeframes. Given the shared climate and health trends, as well as the interconnected socio-economic,	Despite the development of NAPs, health impacts are often addressed in isolation, resulting in gaps between policy and practice. South Africa's comprehensive H-NAP contrasts with those of other countries, where health impacts are less prioritized. It is

									the effects of climate change.	documentation was found online. All five countries referenced having an adaptation policy and indicated that implementation plans were being developed.	environmental, and political landscape, there is significant potential for regional coordination to address cross-border climate change impacts and optimize resource use. SADC member states have begun efforts to strengthen monitoring and development. Key initiatives include the Infectious Disease Warning Programme, Monitoring for Environment and Security in Africa, and Climate for Development in Africa, which aim to enhance access to and use of earth observation data for policy development and collaboration. Updating SADC health frameworks and implementation plans could further strengthen these synergies. Additionally, regional leadership should coordinate efforts to address health-related climate issues,	crucial to address the disconnect between policy scope and practice, particularly by integrating the health impacts of conflict and fragility. Challenges related to the development and implementation of climate adaptation strategies are especially evident in countries, such as those in the Southern African sub-region, that exhibit high vulnerability and low adaptive capacity to the effects of climate variability. The SADC region's heavy reliance on agriculture and multiple stressors exacerbates the health impacts of climate change, including heightened food and water insecurity, as well as the increased prevalence of water- and vector-borne diseases. Despite these challenges, health considerations are often under-prioritized, and

											improve regional and national skills, and address resource gaps.	implementation plans remain fragmented
